# Efficacy of Crizotinib After Entrectinib Resistance Due to *MET* Polysomy in *ROS1*-Rearranged NSCLC: A Case Report

**DOI:** 10.1016/j.jtocrr.2023.100523

**Published:** 2023-05-04

**Authors:** Toshiaki Takakura, Hiroaki Kanemura, Kazuko Sakai, Kazuto Nishio, Kazuhiko Nakagawa, Hidetoshi Hayashi

**Affiliations:** a Department of Medical Oncology, Kindai University Faculty of Medicine, Osaka, Japan; b Internal Medicine III, Wakayama Medical University, Wakayama, Japan; c Department of Genome Biology, Kindai University Faculty of Medicine, Osaka, Japan

**Keywords:** ROS1, *MET* polysomy, Entrectinib, Crizotinib, Non–small cell lung cancer, Case report

## Abstract

Resistance to ROS1 tyrosine kinase inhibitors is inevitable, but it has been unclear whether crizotinib might be effective after the development of entrectinib resistance. We here present a case of *ROS1-*rearranged NSCLC that responded to crizotinib after tumor progression due to *MET* polysomy during entrectinib treatment. This case suggests that crizotinib is an effective option for patients with *MET* polysomy, even after disease progression on entrectinib.

## Introduction

Tumors with *ROS1* rearrangement account for approximately 1% to 2% of NSCLC cases,^1^ and entrectinib and crizotinib have markedly improved outcomes for patients with *ROS1*-rearranged NSCLC. Nevertheless, resistance to entrectinib inevitably develops. The mechanisms of such resistance remain unclear in most cases, and it is not known whether crizotinib is effective after the development of entrectinib resistance. We now report a case of resistance to entrectinib that was characterized by next-generation sequencing (NGS) analysis and overcome by crizotinib.

## Case Presentation

**Figure 1:**
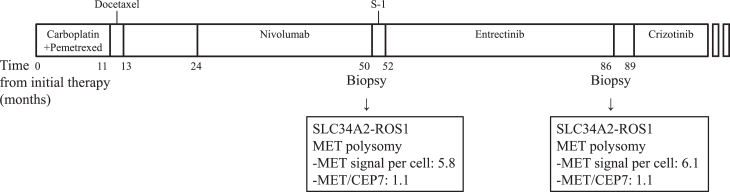
Timeline of the patient’s treatment course and molecular profile. Next-generation sequencing analysis of tumor specimens obtained from the right upper lobe of the lung before and after entrectinib treatment revealed a *ROS1* fusion gene and *MET* polysomy. Neither secondary mutations in the ROS1 kinase domain nor activation of bypass signaling pathways was detected.

A 64-year-old woman who had never smoked presented with dyspnea. A computed tomography (CT) scan revealed a 29-mm nodule in the right upper lobe of the lung, enlargement of the right hilar lymph node and bilateral supraclavicular lymph nodes, and pleural effusion. She was diagnosed with having stage IV lung adenocarcinoma accompanied by malignant pleural effusion. She received first-line treatment with carboplatin plus pemetrexed for four cycles, resulting in a confirmed partial response as the best response (Fig. 1). After 11 months of chemotherapy, a CT scan revealed tumor progression in the right lung. The patient received second-line treatment with docetaxel for four cycles and then again manifested disease progression. A biopsy performed by bronchoscopy for gene testing revealed neither *EGFR* mutation nor *ALK* translocation. She received nivolumab as a third-line treatment and achieved a partial response. After 25 months, a CT scan identified tumor progression in the right lung. S-1 was then administered as a fourth-line treatment for two cycles, after which disease progression was again apparent. A bronchoscopic biopsy from the upper lobe of the right lung was again performed, and the tissue was subjected to NGS (Archer FusionPlex CTL panel), which revealed the presence of an *SLC34A2-ROS1* fusion gene.

**Figure 2:**
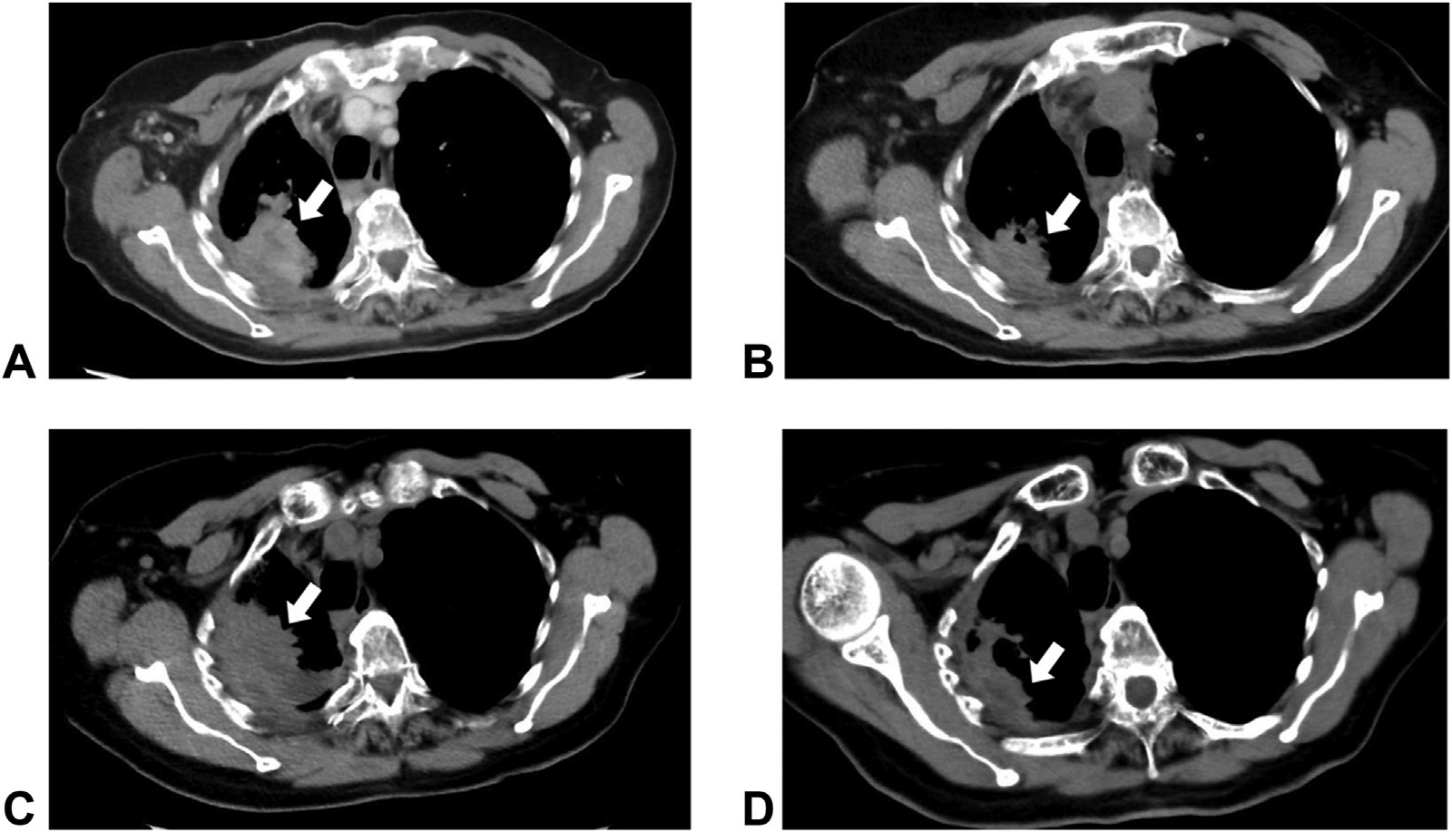
Computed tomography images of the tumor in the right upper lobe of the lung. (A) Before the start of entrectinib treatment. (B) Three months after initiation of entrectinib treatment revealing a size reduction. (C) Before crizotinib treatment. (D) Two months after initiation of crizotinib treatment revealing tumor shrinkage. White arrows indicate the lesions.

The patient received entrectinib (600 mg/d) as a fifth-line treatment, and she achieved a partial response (−30%) in the upper lobe of the right lung at 3 months (Fig. 2). The entrectinib dose was reduced to 400 mg/d as a result of the development of peripheral edema at 9 months. After 34 months of treatment, a CT scan revealed tumor progression in the upper lobe of the right lung. She therefore received crizotinib as a sixth-line treatment, and a CT scan revealed a partial response (−30%) in the upper lobe of the right lung at 2 months.

**Figure 3:**
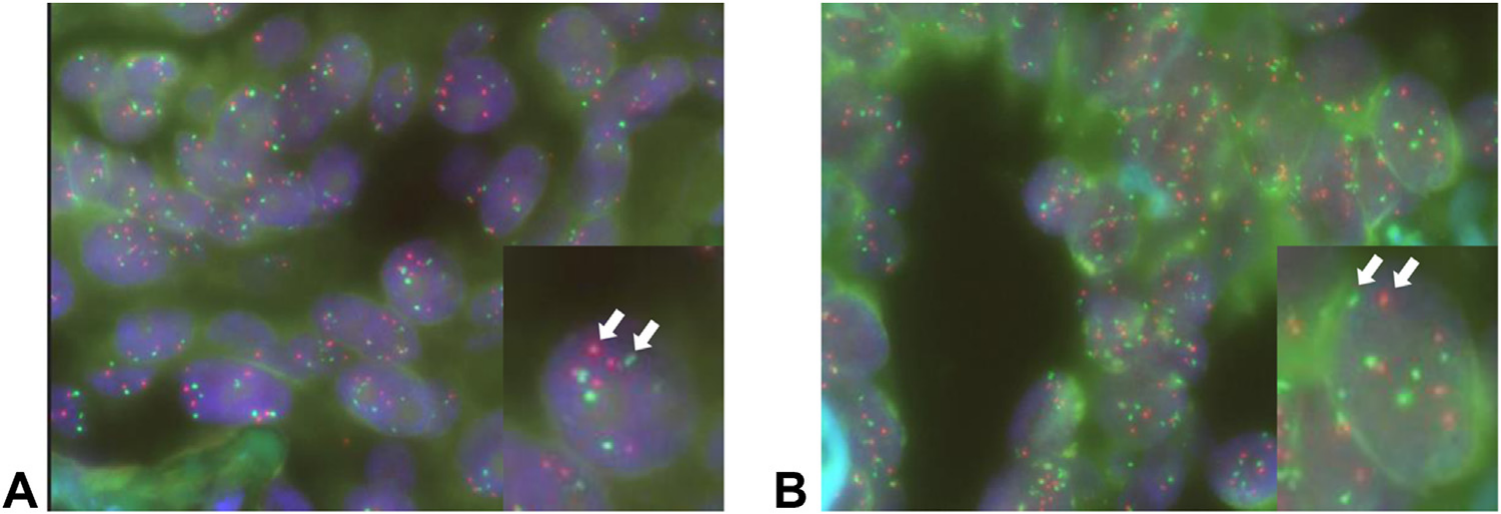
Fluorescence in situ hybridization analysis of *MET*. A low *MET* (red dots)-to-*CEP7* (green dots) ratio but *MET* copy number gain were detected on the basis of the counting of 50 tumor cells (*A*) before and (*B*) after the development of entrectinib resistance. The arrows indicate red and green signals in the cells illustrated at higher magnification in the insets.

We performed NGS analysis of tumor specimens obtained both before and after entrectinib treatment to evaluate the mechanism of crizotinib action. This analysis revealed a *MET* gene copy number of 3 in both specimens, but neither secondary mutations in the ROS1 kinase domain nor activation of bypass signaling pathways was detected. Fluorescence in situ hybridization (FISH) analysis of *MET* amplification revealed *MET* polysomy with probes for *MET* and *CEP7*. The pretreatment specimen revealed 5.8 signals per cell for *MET* and indicated a *MET*-to-*CEP7* ratio of 1.1, whereas the posttreatment specimen revealed 6.1 signals per cell for *MET* and indicated a *MET*-to-*CEP7* ratio of 1.1 (Fig. 3).

## Discussion

As far as we are aware, this is the first report to explore the mechanism of a response to crizotinib in a patient with *ROS1-*rearranged NSCLC after tumor progression during treatment with entrectinib. Indeed, only one case of a crizotinib response after disease progression during entrectinib treatment has been described.^2^ KRAS G12C mutation, *KRAS* amplification, *FGF3* amplification, *MET* amplification, and the *ROS1* mutations G2032R and F2004C/I have been identified as mechanisms of resistance to entrecinib.^3^, ^4^, ^5^, ^6^ Our report suggests that crizotinib could be an effective option for patients with *MET* amplification or polysomy, even after disease progression during entrectinib treatment.

In the present case, although the *MET* gene copy number determined by NGS analysis was 3, FISH analysis revealed a *MET* signal per cell value of approximately 6. An increase in *MET* copy number has been classified on the basis of the *MET* to centromere ratio as polysomy (*MET*-to-centromere ratio of <1.8) or low (>1.8 to <2.2), intermediate (>2.2 to <5.0), or high (>5.0) amplification.^7^ In the FISH assay for the present patient, signals from 50 nonoverlapping nuclei in representative fields were enumerated for *MET* and *CEP7* copy numbers. The analysis revealed *MET* polysomy on the basis of a copy number gain (5.8–6.1 signals per cell) and low ratio of *MET* to *CEP7* (1.1). Both entrectinib and crizotinib are tyrosine kinase inhibitors for ALK and ROS1. In contrast to entrectinib, however, crizotinib does not inhibit TRK but is a potent inhibitor of MET. A patient with NSCLC having a *MET* copy number gain without a high *MET*-to-*CEP7* ratio was previously reported to experience a rapid response to crizotinib.^8^ In addition, a phase 2 study of crizotinib efficacy for NSCLC found that patients with greater than or equal to six copies of *MET* as determined by FISH, including those with *MET* polysomy, had the best objective response rate (32%) and median progression-free survival (3.2 mo).^9^ Indeed, tumor shrinkage was observed in some patients with *MET* polysomy in these previous studies,^7^^,^^9^ suggesting that some patients with *MET* polysomy benefit from crizotinib. *MET* polysomy might therefore have the potential to be an oncogenic driver that is targetable by crizotinib.

Although the current patient had *MET* polysomy before entrectinib, she had an initial response to entrectinib treatment. It has been proposed that intratumoral heterogeneity is associated with a differential drug response.^10^ Although the growth of most cells harboring the *ROS1* rearrangement in the present patient might have been inhibited by entrectinib, a small subpopulation of cells also harboring *MET* polysomy might have survived during continuous entrectinib treatment and become the dominant population in the entrectinib-resistant tumor.

## Conclusions

Our report suggests that crizotinib is an effective option for patients with *MET* amplification or polysomy, even after disease progression during entrectinib treatment. Further study is warranted to understand the mechanisms of entrectinib resistance in *ROS1*-rearranged NSCLC and the efficacy of crizotinib in NSCLC with *MET* polysomy.

## Credit Authorship Contribution Statement

**Toshiaki Takakura**: Writing—original draft, Investigation, Data curation.

**Hiroaki Kanemura, Kazuko Sakai, Kazuto Nishio**: Investigation, Data curation.

**Hidetoshi Hayashi**: Supervision, Writing—review and editing, Conceptualization.
